# Biological and functional characterization of bone marrow-derived mesenchymal stromal cells from patients affected by primary immunodeficiency

**DOI:** 10.1038/s41598-017-08550-5

**Published:** 2017-08-15

**Authors:** Nadia Starc, Daniela Ingo, Antonella Conforti, Valeria Rossella, Luigi Tomao, Angela Pitisci, Fabiola De Mattia, Immacolata Brigida, Mattia Algeri, Mauro Montanari, Giuseppe Palumbo, Pietro Merli, Paolo Rossi, Alessandro Aiuti, Franco Locatelli, Maria Ester Bernardo

**Affiliations:** 1 Department of Pediatric Hematology/Oncology, IRCCS Bambino Gesù Children’s Hospital, Rome, Italy; 2 Department of System Medicine, University of Rome “Tor Vergata”, Rome, Italy; 3 San Raffaele Telethon Institute for Gene Therapy, SR-TIGET; Pediatric Immunohematology, San Raffaele Scientific Institute, Milan, Italy; 4 University Department of Pediatrics, Unit of Immune and Infectious Diseases, IRCCS Bambino Gesù Children’s Hospital, Rome, Italy; 5 Vita-Salute San Raffaele University, Milan, Italy; 6 Department of Pediatrics, University of Pavia, Pavia, Italy

## Abstract

Mesenchymal stromal cells (MSCs) represent a key component of bone marrow (BM) microenvironment and display immune-regulatory properties. We performed a detailed analysis of biological/functional properties of BM-MSCs derived from 33 pediatric patients affected by primary immune-deficiencies (PID-MSCs): 7 Chronic Granulomatous Disease (CGD), 15 Wiskott-Aldrich Syndrome (WAS), 11 Severe Combined Immunodeficiency (SCID). Results were compared with MSCs from 15 age-matched pediatric healthy-donors (HD-MSCs). Clonogenic and proliferative capacity, differentiation ability, immunophenotype, immunomodulatory properties were analyzed. WB and RT-qPCR for CYBB, WAS and ADA genes were performed. All PID-MSCs displayed clonogenic and proliferative capacity, morphology and immunophenotype comparable with HD-MSCs. PID-MSCs maintained the inhibitory effect on T- and B-lymphocyte proliferation, except for decreased inhibitory ability of SCID-MSCs at MSC:PBMC ratio 1:10. While HD- and CGD-MSCs were able to inhibit monocyte maturation into immature dendritic cells, in SCID- and WAS-MSCs this ability was reduced. After Toll-like Receptor priming, PID-MSCs displayed *in vitro* an altered gene expression profile of pro- and anti-inflammatory soluble factors. PID-MSCs displayed lower PPARγ levels and WAS- and SCID-MSCs higher levels of key osteogenic markers, as compared with HD-MSCs. Our results indicate that PID-MSCs may be defective in some functional abilities; whether these defects contribute to disease pathophysiology deserves further investigation.

## Introduction

Mesenchymal stromal cells (MSCs) are adult multipotent cells that can be isolated from multiple tissue sources, including bone marrow (BM)^1, 2^. They can be induced to differentiate *in vitro* into cells of mesodermal lineages^3^, and display unique immunomodulatory properties towards all cells involved in immune response^4–7^. Based on their immune-modulatory properties, MSCs have been used in the treatment of acute graft-versus-host disease (aGvHD) developing after allogeneic hematopoietic stem cell transplantation (HSCT)^8, 9^, and their co-infusion with hematopoietic stem cells (HSCs) has been reported to hasten hematopoietic engraftment after both autologous and allogeneic HSCT^10, 11^. Moreover, given the capacity to modulate immune response and promote tissue repair^12^, MSCs are also being suggested as anti-inflammatory treatment for several autoimmune/inflammatory disorders^13, 14^.

Primary immunodeficiencies (PIDs) represent a heterogeneous group of monogenic conditions, characterized by altered immune responses of innate and/or adaptive immunity^15^. While Wiskott-Aldrich syndrome (WAS) is caused by mutations in the WAS gene expressed in hematopoietic cells^16, 17^, chronic granulomatous disease (CGD) is due to defects in genes encoding the nicotinamide adenine dinucleotide phosphate oxidase complex, the most frequent being *CYBB* ^18, 19^. Severe combined immunodeficiency (SCID) represent a heterogeneous group of diseases with susceptibility to opportunistic life-threatening infections, being characterized by lack of protective T-, B-, and sometimes NK-cell response to infections^20^. ADA-SCID, which accounts for 15–20% of all SCID cases, is caused by mutations in the ADA gene impairing ADA enzymatic activity^21^. Allogeneic HSCT remains the definitive curative option for most PIDs, being associated with very good clinical outcomes, which is continuously improving over time even employing partially matched family donors, as recently demonstrated^22, 23^. For those patients who do not have an HLA-matched donor, HSC-gene therapy (HSC-GT) has been successfully developed for several PIDs including ADA-SCID, SCID due to γ-chain deficiency, WAS and CGD^24, 25^.

Scarce information on the BM microenvironment and no data on MSCs obtained from PID patients (PID-MSCs) are available in the literature. In particular, whether these latter cells display intrinsic defects and participate to the disease pathophysiology or they develop secondary alterations due to the underlying immunodeficiency has not been investigated so far. The present study aims at investigating phenotypically and functionally PID-MSCs; in particular, we focused our attention on their ability to interact with adaptive and innate immune cells as compared with MSCs isolated from healthy donors (HD-MSCs), and on their ability to differentiate into bone and adipose tissue. The results of this study may turn out to be important for the clinical application of autologous MSCs in the context of HSC-GT with the aim of facilitating engraftment of gene-corrected HSCs.

## Results

### Characterization of the biological properties of BM-derived PID-MSCs

**Figure 1:**
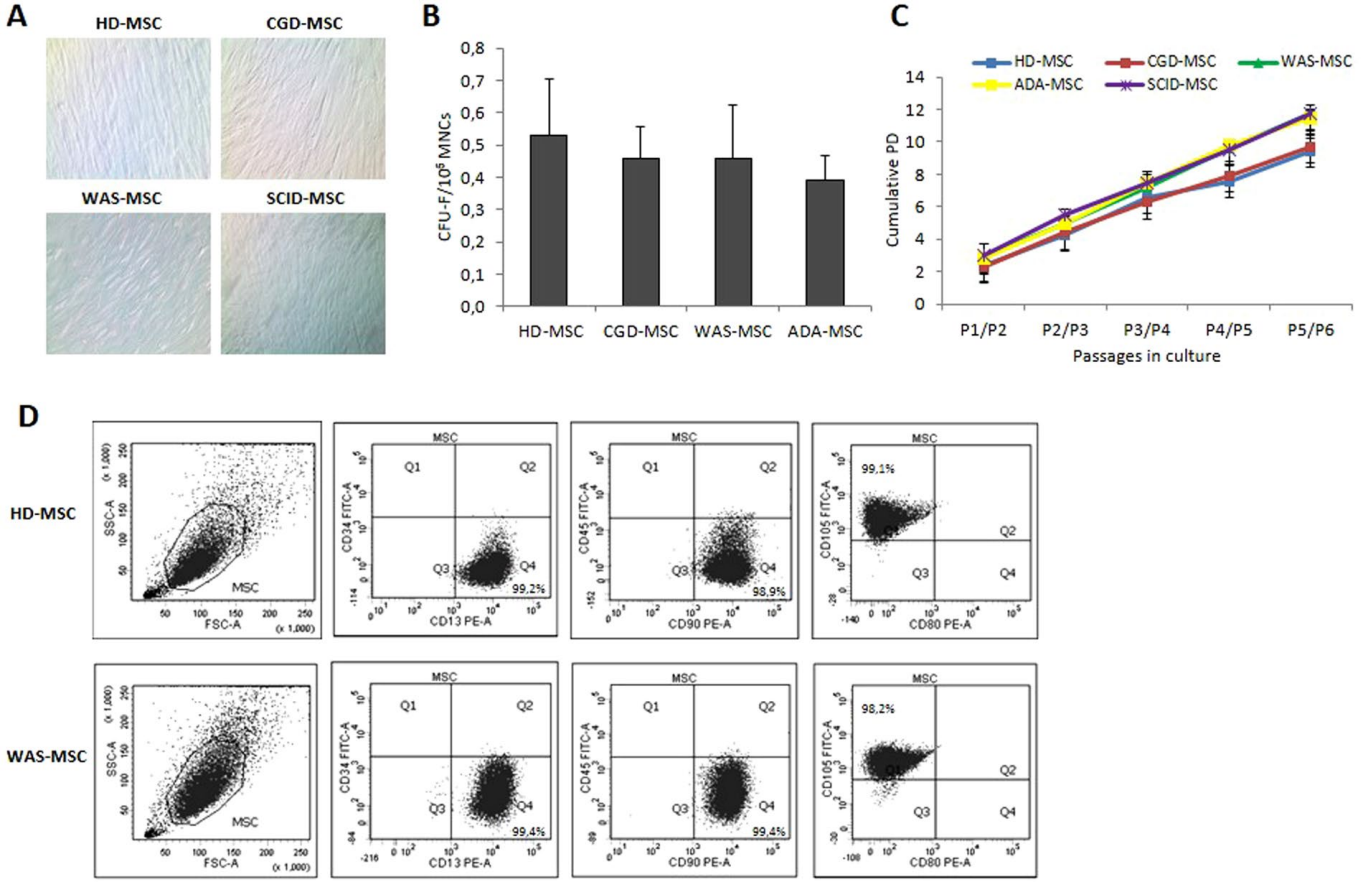
(**A**) Morphology of culture-expanded mesenchymal stromal cells (MSCs) obtained from PID patients (PID-MSCs) and from a pediatric healthy donor (HD-MSCs); a representative example for each disease is shown. MSCs from both patients and donors display the characteristic spindle-shaped morphology (magnification x4). (**B**) Fibroblast-colony forming unit- (CFU-F) ability of PID-MSCs as compared with HD-MSCs. Each bar represents the mean ± SEM of 7 CGD-MSCs, 15 WAS-MSCs, 6 ADA-MSCs and 15 HD-MSCs. CFUs for non-ADA SCID-MSCs were not reported because available only for 2 patients. (**C**) Cumulative population doublings (PDs) from passage (P) 1 to P5 of MSCs isolated from HDs (blue line), CGD- (red line), WAS- (green line), ADA- (yellow line) and SCID-patients (purple line). The data represent the mean (±SEM) of 7 CGD-MSCs, 15 WAS-MSCs, 6 ADA-SCID, 5 non-ADA SCID-MSCs and 15 HD-MSCs. (**D**) Immuno-phenotype of culture-expanded HD-MSCs and PID-MSCs from two representative samples (HD-MSCs in the upper panel and WAS-MSCs in the lower panel). Histograms of surface marker expression are similar: positive for CD13, CD90, CD105 surface antigens and negative for CD34, CD45 and CD80 molecules. Abbreviations: MNCs, mononuclear cells.

MSCs were successfully isolated from BM of all PID patients and propagated *in vitro*; their biological and functional properties were characterized and compared with those of HD-MSCs. As shown in Fig. 1A, all PID-MSCs displayed the characteristic spindle-shaped morphology, similar to that of HD-MSCs. As far as the clonogenic capacity is concerned, PID-MSCs showed a similar CFU efficiency as compared with HD-MSCs (P = NS for each PID-MSC group; Fig. 1B); moreover, they displayed a comparable proliferative capacity with HD-MSCs (P = NS for each PID-MSC group as compared with HD-MSC; see Fig. 1C). All MSC samples were maintained in culture until reaching proliferative senescence, which was observed after a number of passages comprised between P8 and P14 for both HD- and PID-MSCs, this indicating a similar *in vitro* life-span of MSCs (see Supplementary Table 1 for details). Senescent state was confirmed by the positivity for β-galactosidase staining in both cell types (data not shown). Thereafter, PID-MSCs were monitored in culture for up to 6–8 weeks; no changes in morphology and/or proliferation rate, suggestive for neoplastic transformation, were observed. PID-MSCs were immunophenotypically characterized by flow-cytometry at early passages, i.e. P2 or P3. Their phenotype was consistent with that of HD-MSCs previously published^26, 27^; in particular, we analyzed the presence of some hematopoietic markers in order to exclude the contamination of our cultures with other cellular types: CD34 (expressed on hematopoietic stem cells), CD45 (expressed on leukocytes) and CD80 (expressed on dendritic cells) which were no longer detectable by P2, while more than 95% of all PID-MSCs and HD-MSCs expressed the typical surface markers CD13, CD90, CD105 (see Fig. 1D for a representative example from HD- and WAS-MSCs and Supplementary Fig. 1 for the other disease groups). Furthermore, FACS analyses showed no differences in terms of forward scatter and side scatter between PID- and HD-MSCs (see Supplementary Fig. 1).

Gp91phox and WAS proteins and gene expression were analyzed by WB and RT-qPCR in CGD- and WAS-MSCs, respectively. At the protein level, we could not detect Gp91phox neither in HD- nor in CGD-MSCs; these data were confirmed by RT-qPCR, which showed no expression of *CYBB* in patient- and HD-MSCs, in line with its restricted expression in mature monocytes and B-lymphocytes. As far as WAS expression is concerned, WASp could not be evidenced by WB both in WAS- and HD-MSCs and the WAS gene was not expressed by RT-qPCR in both patient and HD-derived cells (data not shown). When ADA enzymatic activity was evaluated, we found lower levels in ADA-MSCs (mean 76.73 nano-moli/h/mg; range 29.97–116.80), as compared with HD-MSCs (mean 1096.38 nano-moli/h/mg; range 532.02–2290.03; P = 0.07). Accordingly, the expression of the ADA gene was reduced in ADA-MSCs, as compared with HD-MSCs (P = NS) (see Supplementary Fig. 2).

### Characterization of the functional properties of BM-derived PID-MSCs

#### Inhibition of T- and B-cell function by PID-MSCs

In order to evaluate the interaction between PID-MSCs and T cells, we measured PBMC proliferation in response to PHA either in the presence or in the absence of PID-MSCs in an allogeneic setting and compared the results with those obtained with HD-MSCs.

**Figure 2:**
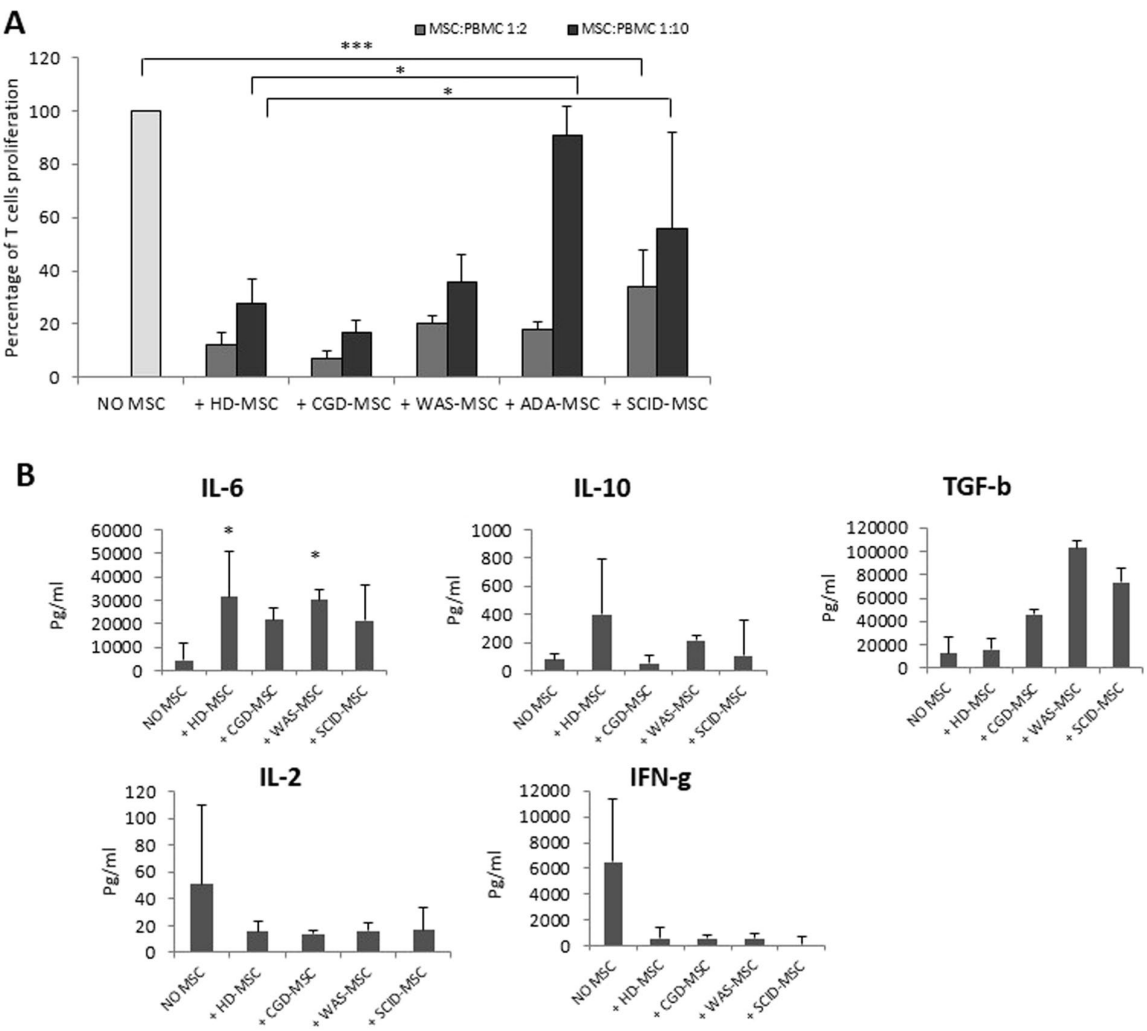
(**A**) *In vitro* immunomodulatory effect of HD- and PID-MSCs on healthy donor (HD) peripheral blood mononuclear cells (PBMCs) in an allogeneic setting. The graph shows the percentage of residual proliferation of HD-PBMCs stimulated with phytohaemagglutinin (PHA) either in the absence (NO MSC) or in the presence of HD- (+HD-MSC), CGD- (+CGD-MSC), WAS- (+WAS-MSC), ADA- (+ADA-MSCs) and SCID-MSCs (+SCID-MSC). Each bar represents the percentage of residual proliferation of 10^5^ PBMCs, in the presence of two different MSC:PBMC ratios (MSC:PBMC ratio of 1:2 and 1:10), calculated by measuring 3H-thymidine incorporation after 72 hours co-culture. We referred to PBMC proliferation alone (in the absence of MSCs) as 100% and this percentage was used to normalize PBMC proliferation in the presence of MSCs. Mean ± SEM of multiple experiments performed at least twice on 9 HD-MSCs, 7 CGD-MSCs, 12 WAS-MSCs, 6 ADA-MSCs and 5 SCID-MSCs (each point being in triplicate) is reported. P values lower than 0.05 were considered to be statistically significant (*p < 0.05; ***p < 0.001). (**B**) Levels of anti-inflammatory and pro-inflammatory cytokines in supernatants of co-cultures of 3 HD-MSCs, 3 WAS-MSCs, 2 CGD-MSCs and 2 SCID-MSCs (RAG-1 deficiency) with PBMCs, after 72-hour incubation with PHA. Results are expressed as pg/ml of the mean ± SEM. P values lower than 0.05 were considered to be statistically significant (*p < 0.05).

In line with previously reported data^28^, HD-MSCs showed *in vitro* a significant inhibitory capacity on PHA-induced PBMC proliferation, which was dose-dependent (P < 0.001 as compared with PBMCs + PHA alone, for both 1:2 and 1:10 MSC:PBMC ratios). As far as PID-MSCs are concerned, both CGD-MSCs and WAS-MSCs exerted strong *in vitro* inhibitory effect on T-lymphocyte proliferation which was similar to that of HD-MSCs (P = NS for both ratios). Notably, while ADA-MSCs and SCID-MSCs were able to significantly inhibit PBMC proliferation at MSC:PBMC ratio 1:2 (P < 0.001 as compared with PBMCs + PHA alone and P = NS as compared with HD-MSCs at the same ratio), they both showed a decreased inhibitory ability at the MSC:PBMC ratio 1:10 (P = NS as compared with PBMCs + PHA alone and P < 0.05 as compared with HD-MSCs at the same ratio for both ADA- and SCID-MSCs), which was more evident for ADA-MSCs (see Fig. 2A).

In order to better understand the mechanisms underlying MSC immune-regulatory ability on T cells, pro- and anti-inflammatory cytokines (*i*.*e*. IFN-γ, TGF-β, IL-2, IL-6, IL-10) were measured in MSC:PBMC co-cultures (1:2 ratio) stimulated with PHA for 3 HD-MSC samples, 3 WAS-MSC samples, 2 CGD- and 2 SCID-MSCs (RAG-1 deficiency) samples. In agreement with previous publications^29^, in the presence of HD-MSCs the levels of anti-inflammatory cytokines (IL-6, IL-10 and TGF-β) increased; a similar behavior was observed when PID-MSCs were added to the co-cultures. In particular, the amount of IL-6 detected was significantly increased in PHA stimulated PBMCs co-cultured in the presence of HD-MSCs (31 ng/ml, SEM ± 19; P < 0.05) and WAS-MSCs (30.1 ng/ml, SEM ± 4.5; P < 0.001), as compared with PHA stimulated PBMCs alone (4.5 ng/ml, SEM ± 7); by contrast, in the presence of SCID-MSCs and CGD-MSC only a trend for superior levels of this cytokines was observed (P = NS as compared with the condition without MSCs). An increase in IL-10 levels, although not statistically significant, was observed in the presence of both HD- and PID-MSCs as compared with the condition without MSCs (P = NS), the only exception being CGD-MSCs where the levels of IL-10 were lower as compared with the condition without MSCs (P = NS). Moreover, levels of TGF-β were higher in the presence of each PID-MSCs, although the P values were not statistically different as compared with HD-MSCs. The levels of pro-inflammatory cytokines (IL-2 and IFN-γ) decreased in MSC:PBMC co-cultures from both HD- and PID-MSCs, as compared with PHA-stimulated PBMCs. In case of WAS-MSC co-cultures, the amount of IFN-γ was significant lower (597 pg/ml, SEM ± 370; P < 0.01), as compared with PHA stimulated PBMCs alone (6479 pg/ml, SEM ± 4920; see Fig. 2B); statistics for IL-2 and IFN-γ in the other disease groups could not be performed due the limited number of samples available for analysis.

**Figure 3:**
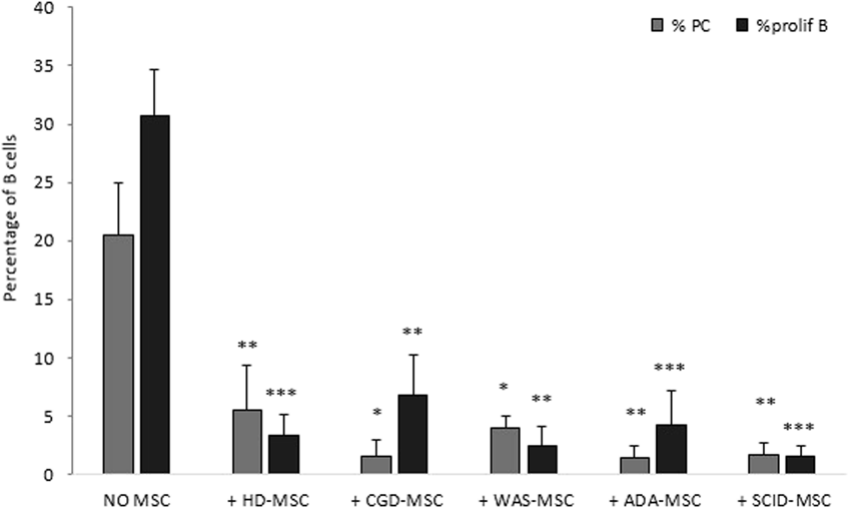
*In vitro* immunomodulatory effect of HD- and PID-MSCs on B-cell proliferation and plasma-cell (PC) differentiation in an allogeneic setting. PBMCs were stimulated with CpG for 7 days and co-cultured with or without MSCs. Cells were collected, stained for CMFDA, CD19, CD27, CD38 and IgM and analyzed by flow-cytometry. The graph shows the percentage of proliferating B cells and plasma-cells stimulated with CpG either in the absence (NO MSC) or in the presence of HD-, CGD-, WAS-, ADA- and SCID-MSCs (+HD-MSC, +CGD-MSC, +WAS-MSC, +ADA-MSCs, +SCID-MSC) (MSC:PBMC ratio 1:10). Each bar represents the mean ± SEM of multiple experiments (9 HD-MSCs, 5 CGD-MSCs, 9 WAS-MSCs, 4 ADA-MSCs and 4 SCID-MSCs were tested at least twice and each point being in triplicate). P values lower than 0.05 were considered to be statistically significant (*p < 0.05; **p < 0.01; ***p < 0.001).

In order to assess the immune-regulatory effect of PID-MSCs on B lymphocytes, we measured B-cell proliferation and plasma-cell generation after 7-day MSC:PBMC co-culture stimulated with CpG in an allogeneic setting. Both HD- and PID-MSCs were able to significantly inhibit B-cell proliferation and plasma-cell generation, as compared with PBMCs + CpG alone (P < 0.05 for CGD- and WAS-MSCs and P < 0.01 for HD-, ADA- and SCID-MSCs) (see Fig. 3). Moreover, no statistically significant differences were found in the ability to influence B-cell functionality between PID-MSCs and HD-MSCs (P = NS for the 4 disease groups).

#### Inhibitory effect of PID-MSCs on monocyte maturation

**Figure 4:**
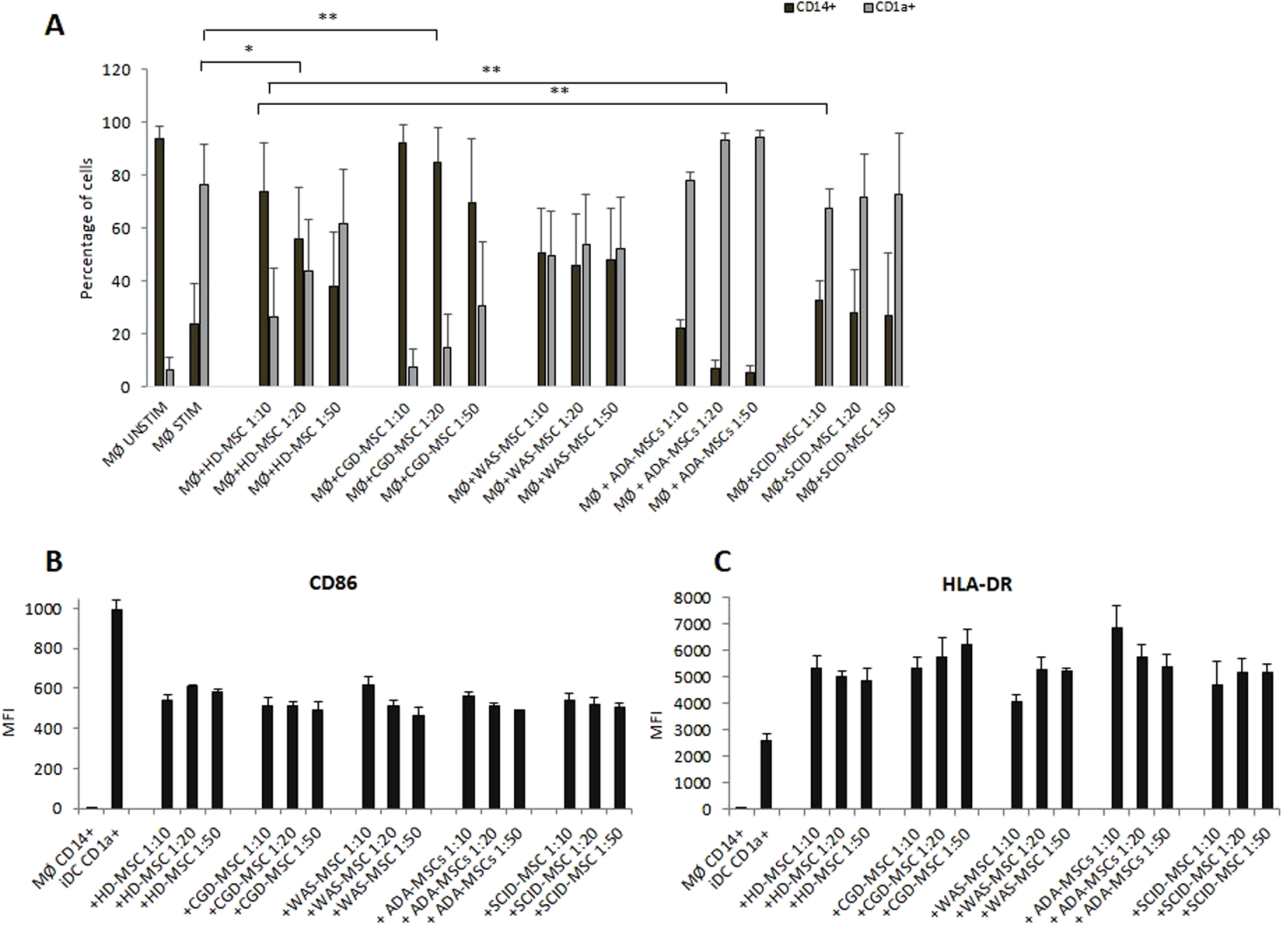
*In vitro* immunomodulatory effect of HD- and PID-MSCs on monocyte (MØ) maturation into immature dendritic cells (iDCs) isolated from HDs. (**A**) The graph shows the percentage of MØ (CD14^+^, black bars) and DCs (CD1a^+^, grey bars) co-cultured in the presence or in the absence of MSCs in a medium additioned with interleukin 4 (IL-4) and granulocyte macrophage-colony stimulating factor (GM-CSF). Each bar represents the percentage of residual proliferation of 10^5^ MØ, in the presence of three different MSC:MØ ratios (MSC:MØ ratio of 1:10, 1:20 and 1:50). After 7-day co-culture, cells were collected, stained for CD14 and CD1a and analyzed by flow-cytometry. Mean ± SEM of multiple experiments (7 HD-MSCs, 7 CGD-MSCs, 8 WAS-MSCs, 5 ADA-MSCs and 5 SCID-MSCs were tested at least twice and each point being in triplicate) is reported. P values lower than 0.05 were considered to be statistically significant (*p < 0.05; **p < 0.01). (**B**,**C**) Expression of CD86 (B) and HLA-DR (C) on MØ co-cultured in the presence and in the absence of MSCs in a medium additioned with IL-4 and GM-CSF, evaluated as MFI (mean fluorescence intensity) for 3 HD-MSCs, 3 CGD-MSCs, 3 WAS-MSCs, 3 ADA-MSCs and 3 SCID-MSCs samples. Each bar represent the mean ± SEM.

To investigate whether PID-MSCs maintain the ability to modulate also innate immune responses, we focused on MSC effect on differentiation and maturation of monocyte-derived dendritic cells (DCs). Both HD- and PID-MSCs were cultured in transwell inserts with peripheral blood-derived CD14^+^ monocytes in the presence of IL-4 and GM-CSF, cytokines known to be able to promote the differentiation into immature CD1a^+^ DCs (iDCs). We observed that both HD- and CGD-MSCs were able to efficiently inhibit iDCs generation; in particular, the mean percentage of CD14^+^ cells after co-culture with MSCs at MSC:Monocyte ratio 1:10 was 74% (SEM ± 18; P < 0.05 as compared with stimulated monocytes in the absence of MSCs) for HD-MSCs and 92% (SEM ± 6.7; P < 0.01 as compared with stimulated monocytes in the absence of MSCs) for CGD-MSCs. ADA- and SCID-MSCs displayed a significantly reduced ability to inhibit iDCs differentiation as compared with HD-MSCs with a mean percentage of CD14^+^ cells after co-culture of 22% (SEM ± 3.5; P < 0.01 as compared with HD-MSCs) for ADA-MSCs, and 33% (SEM ± 7; P < 0.01 as compared with HD-MSCs) for SCID-MSCs at MSC:Monocyte ratios 1:10. WAS-MSCs showed an intermediate behavior between CGD- and the 2 groups of SCID-MSCs with a mean percentage of CD14^+^ cells after co-culture of 50.5% (SEM ± 17; P = NS as compared with stimulated monocytes in the absence of MSCs) (see Fig. 4A). These experiments were performed also at MSC:Monocyte ratios 1:20 and 1:50 and showed similar and dose-dependent results as compared with the ratio 1:10 (see Fig. 4A).

Furthermore, for a more meaningful characterization of MSC effect on the differentiation of monocytes into DCs, we looked also at the expression of HLA-DR and CD86 after 7-day co-culture. We found that, in the absence of MSCs and in presence of GM-CSF and IL-4, CD1a^+^ cells expressed some levels of both HLA-DR and CD86, as reported for classic iDCs^30, 31^. In line with data published da Gao *et al*.^30^, after co-culture with both HD- and PID-MSCs, monocytes displayed a lower, although not statistically different, expression of CD86 at all MSC:Monocyte ratios, as compared with monocytes stimulated alone (P = NS; see Fig. 4B). The levels of HLA-DR tended to increase in the presence of MSCs, both from HDs and PIDs, although the difference was not statistically significant as compared with monocytes stimulated alone (P = NS for all MSCs sample) (Fig. 4C). In this case, no differences were found between HD- and CGD-MSCs and SCID- and WAS-MSCs.

#### Effect of priming with TLR3 and TLR4 agonists on immune-regulatory genes expression in PID-MSCs

**Figure 5:**
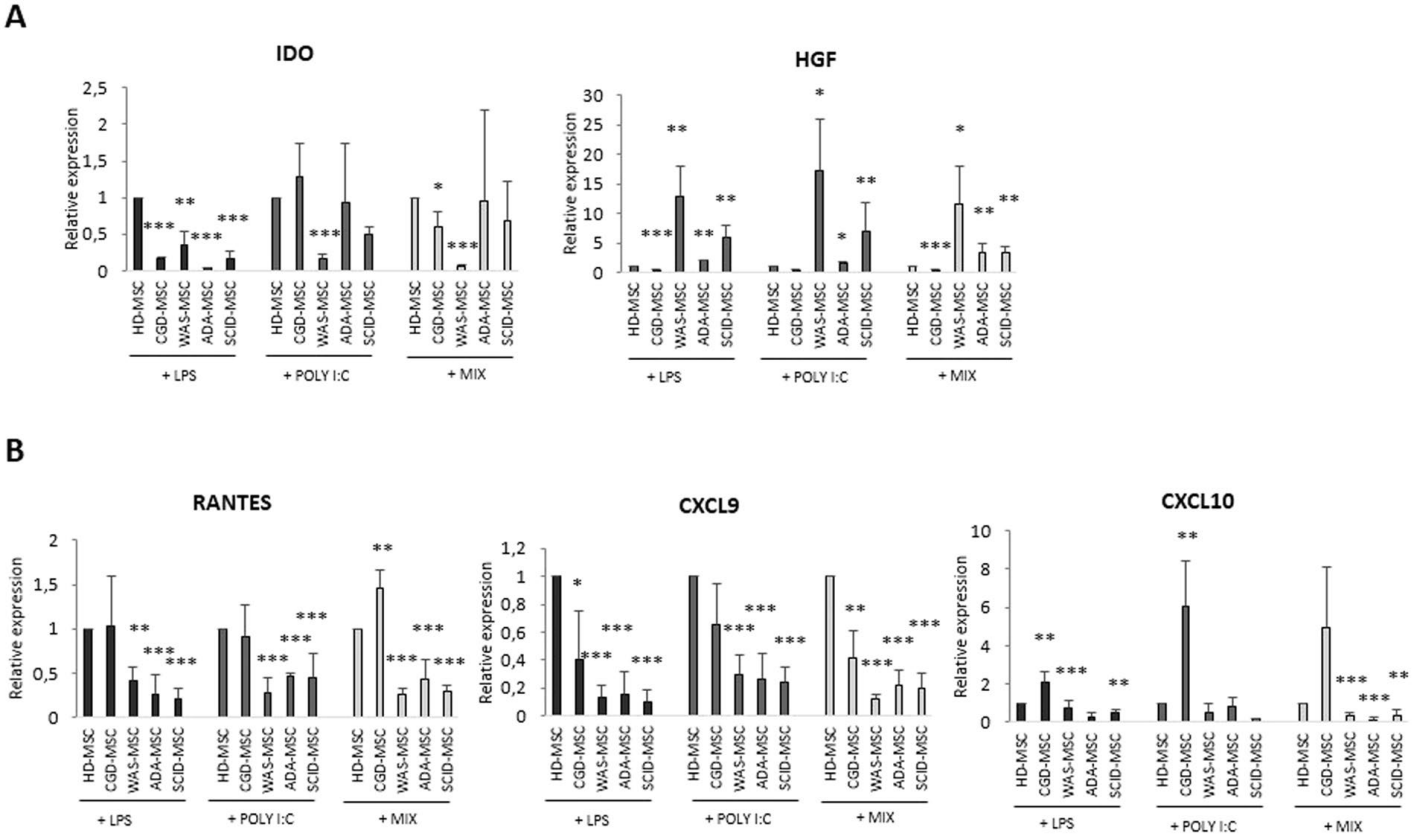
Gene expression of anti- and pro-inflammatory cytokines on HD- and PID-MSCs after TLR3 and TLR4-priming. (**A**) mRNA levels of anti-inflammatory cytokines IDO and HGF in HD- and PID-MSCs 18 hours after treatment with LPS (10 ng/ml), poly I:C (1 μg/ml) or the combination LPS + poly I:C (MIX) were quantified by the 2^−ΔΔCt^ method after normalization with respect to GAPDH. Results are expressed as fold-change relative to HD-MSCs. (**B**) mRNA levels of pro-inflammatory cytokines RANTES, CXCL9 and CXCL10 after treatment with LPS (10 ng/ml), poly I:C (1 μg/ml) or the combination LPS + poly I:C (MIX). Each bar represents the mean ± SEM of multiple experiments (each point was performed in duplicate and repeated independently at least 3 times for 6 HD-MSCs, 6CGD-MSCs, 7 WAS-MSCs, 6 ADA-MSCs and 5 SCID-MSCs sample). P values lower than 0.05 were considered to be statistically significant (compared with HD-MSCs: *p < 0.05; **p < 0.01; ***p < 0.001).

Previous studies have demonstrated that MSCs express several TLRs and that stimulation with specific TLR agonists is able to polarize MSCs into a pro-inflammatory phenotype (MSC1) or an immunosuppressive one (MSC2)^32, 33^. In order to better understand MSC ability to be activated by the surrounding microenvironment in diseases such as PIDs in which severe infections and inflammation are frequently present, we analyzed the effects of TLR signaling on PID-MSCs, as compared with HD-MSCs. To this aim, we performed gene expression analysis of typical MSC immune-regulatory molecules after PID-MSC stimulation with LPS, poly I:C or the combination of LPS + poly I:C. Our results indicate that PID-MSCs display *in vitro* an altered gene expression profile of pro- and anti-inflammatory soluble factors after TLR stimulation (see Fig. 5A,B for anti- and pro-inflammatory soluble factors, respectively). In particular, as far as CGD-MSCs are concerned, gene expression analysis showed that after TLR4-priming, CGD-MSCs displayed significantly higher levels of CXCL10 (2.1-fold increase, SEM ± 0.5; P < 0.01), as compared with HD-MSCs. When the mixture of LPS and poly I:C was employed to challenge CGD-MSCs, both Rantes and CXCL10 increased.

When WAS-MSCs were stimulated through TLR-3 or with the mixture LPS + poly I:C, we observed a significant decrease in IDO levels as compared with HD-MSCs (0.16-fold increase, SEM ± 0.07; P < 0.001) and a significant increase in HGF levels (17.1-fold increase, SEM ± 8; P < 0.05 as compared with HD-MSCs). Meanwhile, TLR-4 stimulation and the mixture LPS + poly I:C induced a significant decrease in the expression of pro-inflammatory cytokine genes (P < 0.01 for Rantes, P < 0.001 for CXCL9, P = NS for CXCL10) in WAS-MSCs. With regard to ADA- and SCID-MSCs, when TLR-3 was primed and the mixture LPS + poly I:C was employed, HGF expression significantly increased as compared with HD-MSCs (1.5-fold increase, SEM ± 0.3, P < 0.05 for ADA-MSCs and 6.8-fold increase, SEM ± 4, P < 0.01 for SCID-MSCs with poly I:C stimulation; 3.3-fold increase, SEM ± 1.5, P < 0.01 for ADA-MSCs and 3.3-fold increase, SEM ± 0.9, P < 0.01 for SCID-MSCs with the mixture LPS + poly I:C); by contrast, TLR-4 stimulation and the mixture LPS + poly I:C were associated with significantly reduced expression of pro-inflammatory molecules for both ADA- and SCID-MSCs, as compared with HD-MSCs (P < 0.001 for Rantes, CXCL9 and CXCL10).

#### PID-MSCs ability to differentiate into osteoblasts and adipocytes

**Figure 6:**
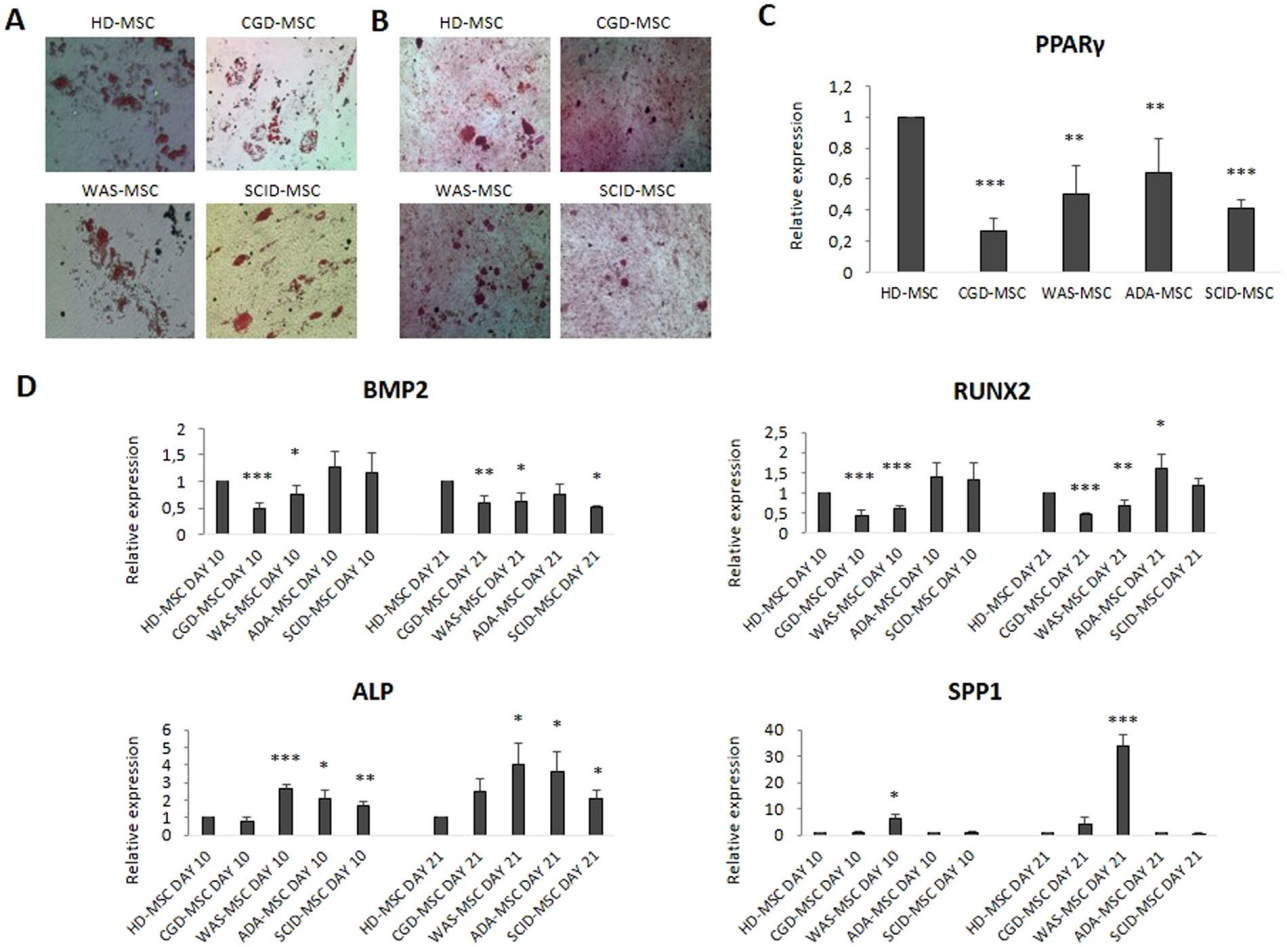
Multi-lineage differentiation potential of HD- and PID-MSCs after 10- and 21-day culture in adipogenic and osteogenic medium. In (**A**) the differentiation into adipocytes is shown by the formation of lipid droplets stained with Oil Red O, magnification x20; in (**B**) the differentiation into osteoblasts is demonstrated by the histological detection of calcium depositions positive for Alizarin Red, magnification x20. (**C**,**D**) Gene expression of key adipogenic marker PPARγ (C) and key osteogenic markers BMP2 (early), RUNX2 (early), ALPL (late) and SPP1 (late) (D) on HD- and PID-MSCs after culture in differentiation medium. mRNA levels of the adipogenic marker were quantified on day +21 of the culture, whereas mRNA levels of osteogenic markers were quantified both on day +10 and on day +21 of the culture to reveal the expression of both early and late osteogenic genes by the 2^−ΔΔCt^ method after normalization with respect to GAPDH. Results are expressed as fold-change relative to HD-MSCs. Each bar represents the mean ± SEM of multiple experiments (each point was performed in duplicate and repeated independently at least 3 times for 7 HD-MSC, 5 CGD-MSC, 6 WAS-MSC, 5 ADA-MSC and 5 SCID-MSC sample). P values lower than 0.05 were considered to be statistically significant (compared with HD-MSCs: *p < 0.05; **p < 0.01; ***p < 0.001).

In order to assess the differentiation capacity of PID-MSCs in comparison with HD-MSCs, cells were induced to differentiate in osteoblasts and adipocytes and analyzed by histological staining. As shown in Fig. 6, all PID-MSCs stained positive with Oil Red O, which reveales the formation of lipid droplets (Fig. 6A); moreover, they showed calcium depositions positive for Alizarin Red (see Fig. 6B) and responded to alkaline phosphatase (ALP) reaction with Fast Blue (not shown).

Gene expression of key adipogenic and osteogenic (early and late) markers was also analyzed. After 21-day culture in conditioned and non-conditioned medium (as control) both HD- and PID-MSCs were able to differentiate into adipocytes. The relative fold increase of PPARγ in conditioned MSCs, as compared with non-conditioned MSCs, was 19.2 (SEM ± 3.5; P < 0.001) for HD-MSCs, 7.6 (SEM ± 2; P < 0.01) for ADA-MSCs, 10.9 (SEM ± 3; P < 0.01) for SCID-MSCs, 13.1 (SEM ± 3.4; P < 0.01) for CGD-MSCs and 16.8 (SEM ± 5.5; P < 0.01) for WAS-MSCs. However, when PPARγ expression in conditioned PID-MSCs was compared with that of conditioned HD-MSCs, PID-MSCs showed a significantly reduced level of the gene; in particular, the relative fold increase for ADA-MSCs was 0.64 (SEM ± 0.2; P < 0.01), SCID-MSCs was 0.41 (SEM ± 0.05; P < 0.001), for CGD-MSCs 0.26 (SEM ± 0.08; P < 0.001) and for WAS-MSCs 0.5 (SEM ± 0.2; P < 0.01) (see Fig. 6C). The early osteogenic markers BMP2 and RUNX2 and the late osteogenic markers ALP and SPP1 were both analyzed at day +10 and +21 of the culture; interestingly, we observed that the expression of RUNX2 and ALP in ADA-MSCs and SCID-MSCs and of ALP and SPP1 in WAS-MSCs was significantly increased, as compared with HD-MSCs. In particular, ADA-MSCs displayed a 1.6-fold increase (SEM ± 0.3; P < 0.05) for RUNX2, a 3.6-fold increase (SEM ± 1.1; P < 0.05) for ALP at day +21, and SCID-MSCs displayed a 2-fold increase (SEM ± 0.5; P < 0.05) for ALP at day +21, whereas WAS-MSCs showed a 4-fold increase (SEM ± 1.2; P < 0.05) for ALP at day +21 and a 33.7-fold increase (SEM ± 4.6; P < 0.001) for SPP1 at day +21, as compared with HD-MSCs (see Fig. 6D). On the contrary, CGD-MSCs seemed to have reduced ability to differentiate into osteoblasts especially in terms of expression of early markers, as demonstrated by the reduced expression of BMP2 (0.5- fold increase, SEM ± 0.1 at day +10, P < 0.001) and RUNX2 (0.4- fold increase, SEM ± 0.1 at day +10, P < 0.001), as compared with HD-MSCs (see Fig. 6D).

## Discussion

With the aim of extensively characterizing their *in vitro* biological and functional properties, we isolated and expanded *ex*-*vivo* BM-derived MSCs from patients affected by PIDs. For a more meaningful interpretation of data, we compared PID-MSCs results with those of their HD-derived counterparts.

Our data demonstrate that MSCs can be consistently generated and expanded *ex*-*vivo* from PID patients. PID-MSCs are morphologically similar to HD-MSCs and express the combination of surface markers commonly employed for identifying BM-derived HD-MSCs^26^. When the clonogenic and proliferative abilities of PID-MSCs were analyzed and compared with those of HD-MSCs, we found that they did not differ (Fig. 1). Moreover, PID-MSCs displayed a life-span *in vitro* similar to that of HD-MSCs and regularly entered into replicative senescence. These data indicate that, when a congenital defect of the immune system is present, MSCs, as fundamental components of the HSC supportive niche, do not seem to display intrinsic defects in these *in vitro* biological characteristics.

Neither HD- nor PID-MSCs expressed CYBB and WAS at the protein and gene levels, in line with data on hematopoietic cells, where these genes are preferentially expressed by mature cells and very low o not expressed by hematopoietic progenitors^34, 35^. For what concerns ADA-MSCs, the expression of the ADA gene, which is known to be ubiquitously expressed, was found to be reduced as compared with HD-MSCs; accordingly, also the levels of ADA enzymatic activity were lower in ADA-MSCs as compared with HD-MSCs.

We showed that PID-MSCs retain immune-regulatory properties on adaptive immunity typical of HD-MSCs, being able to inhibit both T- and B-cell proliferation, as well as plasma-cell generation (Figs 2 and 3). This preserved functionality *in vitro* of PID-MSCs might support the hypothesis of a non-direct involvement of mesenchymal progenitors in the pathophysiology of these diseases, although we cannot exclude that primary PID-MSCs *in vivo* may behave differently. Only ADA-MSCs and SCID-MSCs displayed a significantly reduced ability to inhibit T-cell proliferation when tested at the MSC:PBMC ratio 1:10, which was almost completely abolished for ADA-MSCs. We also evaluated the ability of MSCs to influence some aspects of the innate immunity by studying their capacity to modulate monocyte functions, as it has been reported that HD-MSCs are able to inhibit differentiation and maturation of monocyte-derived DCs^31, 33, 36^. We observed that both HD- and CGD-MSCs were able of efficiently preventing differentiation of monocytes into iDCs in a dose-dependent manner, whereas WAS-, ADA- and SCID-MSCs displayed a reduced ability, which was very evident in SCID-MSCs and completely abolished in ADA-MSCs (Fig. 4A). The impaired capacity of ADA- and SCID-MSCs to manage T-cell responses at high MSC:PBMC ratios and to inhibit differentiation of iDCs might be explained by the lack of stimuli by T/B and, in some cases also NK cells, in the BM microenvironment of these patients, due to their underlying immune defect. In case of ADA-MSCs, these reduced functional abilities might also be directly attributed, at least in part, to the impaired enzymatic activity in ADA-MSCs, as well as to their reduced expression of the ADA gene.

Moreover, we analyzed the expression level of pro- and anti-inflammatory molecules after priming of PID-MSCs with TLR agonists, as a model to test the ability of MSCs to respond to an inflamed microenvironment. In fact, Waterman *et al*.^32^, reported that MSCs are able to polarize into two distinct phenotypes following specific TLR stimulation, the pro-inflammatory MSC1 and the anti-inflammatory MSC2 phenotypes, resulting in different immunomodulatory effects and secretomes. The short incubation time that we employed and the minimal TLR agonist concentrations used are intended to mimic the gradient of danger signals that endogenous MSCs may encounter in the surrounding microenvironment, especially in clinical situations in which infections and/or inflammation are frequently present, such as in PID patients^29^. Comparison of unstimulated MSCs, derived from PID patients and HDs, versus both MSC types primed with TLR agonists indicate that all MSCs were activated, after the challenge, to synthetize chemokines/soluble factors involved in inflammation, independently from the patient or donor origin. However, when PID-MSCs were compared with HD-MSCs, we found that patient-derived cells displayed an altered gene expression profile *in vitro* of both pro- and anti-inflammatory soluble factors. This altered secretory phenotype may reflect an impaired ability of PID-MSCs to sense and respond to the surrounding microenvironment. Indeed, the frequent ongoing infections and extensive inflammation present in PID patients may influence and modify the basal activation status of MSCs, ranging from hyper-activation with increased secretion of soluble factors to exhaustion of their secretory ability and reduced molecule production. For example, a higher expression of the anti-inflammatory factor HGF in WAS-, ADA- and SCID-MSCs may reflect the attempt of MSCs to counterbalance the underlying inflammatory situation with the aim to re-establish tissue homeostasis^37^. On the other hand, the contemporary high levels of the pro-inflammatory molecule CXCL10 in TLR3-primed CGD-MSCs may indicate their inability to handle the hyper-inflammatory status typically present in CGD patients. In light of this altered secretory phenotype, also the absent/impaired ability of ADA-, SCID- and WAS-MSCs to influence maturation of monocytes may be due to modifications in PID-MSC ability to sense the surrounding microenvironment and to respond by producing proper amounts of soluble factors.

We also tested PID-MSCs ability to differentiate *in vitro* into adipocytes and osteoblasts. While histological staining for both adipose and bone tissue resulted positive for all PID-MSCs, when RT-PCR for key differentiation markers was performed we found differences between HD- and PID-MSCs. In particular, we found that PPARγ expression was significantly reduced in MSCs obtained from all patients, as compared with HD-MSCs, indicating an impaired ability to form adipose tissue. As regards the osteogenic differentiation, while CGD-MSCs seemed to display a reduced ability to form bone, as compared with HD-MSCs, WAS-, ADA- and SCID-MSCs expressed significantly higher levels of some osteogenic markers (ALP and SPP1 for WAS-MSCs; BMP2, RUNX2 and ALP for ADA- and SCID-MSCs), as compared with HD-MSCs (Fig. 6). HGF, which is a nutrient factor secreted by MSCs and important for tissue development and regeneration, has been reported to promote *in vitro* osteogenic differentiation of human MSCs^38^ and to stimulate MSC-mediated osteogenic regeneration^39^. Based on these observations, the increased expression of HGF that we have found in SCID-, ADA- and WAS-MSCs may correlate with their increased capacity to differentiate into osteoblasts. Meanwhile, the reduced ability of CGD-MSCs to express early bone markers, together with their altered secretory phenotype, may be explained by the impairment in some of their functional abilities, in line with inherent defects also in the HSC compartment in this disease. Indeed, persistent chronic inflammation in CGD patients has been reported to be associated with hematopoietic proliferative stress and consequent decreased functional activity of their HSCs^40^.

In conclusion, our study provides a comprehensive characterization of BM-derived MSCs obtained from PID patients before allogeneic transplantation/HSC-GT procedure. Our results indicate that PID-MSCs, which represent a key component of the HSC niche, maintain some of the main biological properties of HD-MSCs, as well as the characteristic ability to modulate adaptive immune responses. For what concerns the ability to interact with innate immunity and to sense and respond to the surrounding microenvironment, PID-MSCs seem to be impaired *in vitro* in some of their functions. Whether these defects reflect an intrinsic alteration of mesenchymal compartment in these diseases, which might also negatively influence the biology and functions of the HSCs residing in the BM niche, or are secondary to the altered microenvironment deserves further investigation. For example, in case of ADA-MSCs, this might at least in part reflect an intrinsic defect due to the fact that the expression of the ADA gene, as well as its specific enzymatic activity, is defective in ADA-MSCs, as compared with HD-MSCs. Indeed, an inherent defect of osteoblast functions and a reduced capacity to support *in vitro* and *in vivo* hematopoiesis has been already reported for the BM microenvironment of ADA-deficient mice^41^. Our observations may have clinical implications for the design of MSC-based supportive strategies in the context of HSC-GT both with the aim to facilitate engraftment of gene-corrected HSCs and in view of the potential need to correct also mesenchymal progenitors, especially in those diseases in which an impaired BM stroma may be present. This concept has been previously highlighted by Jacome *et al*.^42^, who reported that the lentiviral transduction of unselected Fanconi Anemia BM cells could mediate an efficient phenotypic correction of both hematopoietic progenitor cells and MSCs, which are known to be defective in this disease^43^.

## Patients and Methods

### PID patients and healthy donors

Thirty-three children were included in the study: 7 had CGD (median age 5 years, range 8 months–12 years), 15 WAS (median age 3 years, range 6 months–12 years), 6 ADA-SCID (median age 2 year, range 10 months–5 years) and 5 SCID other than ADA (3 RAG-1 deficiency and 2 with γ-chain defect; median age 2 year, range 6 months–3 years). Patients were diagnosed at: (i) Department of Pediatric Haematology-Oncology, Istituto di Ricovero e Cura a Carattere Scientifico (IRCCS) Bambino Gesù Children’s Hospital, Rome; (ii) University Department of Pediatrics, Unit of Immune and Infectious Diseases of IRCCS Bambino Gesù Children’s Hospital, Rome; and (iii) San Raffaele-Telethon Institute for Gene Therapy (SR-TIGET), Pediatric Immunohematology, San Raffaele Scientific Institute, Milano. Patient BM was collected, after obtaining parental informed consent, during diagnostic procedures or central venous line placement according to the San Raffaele Hospital–approved research protocol for patho-physiology studies in immune-deficient patients. All experiments were performed in accordance with relevant guidelines and regulations. MSCs were isolated and expanded *ex*-*vivo* from BM aspirates of patients and from residual BM cells of 15 pediatric HDs (median age 8 years, range 2–15 years), who donated BM for hematopoietic cell transplantation at Bambino Gesù Children’s Hospital, used as controls. Patient and HD characteristics are summarized in Table 1.

**Table 1:** Characteristics of PID patients and HDs at time of MSC isolation.

BM-MSC samples	HD 15	CGD7	WAS 15	ADA 6	SCID 5 (3 RAG-1 deficiency, 2 γ-chain defect)
Median age (range) P value (HD age vs. pts. age)	8 years (2–15) P = NA	5 years (8 m–12 y) P = NS	3 years (6 m–12 y) P = NS	2 years (10 m–5 y) P < 0.05	2 years (6 m–3 y) P < 0.05
Males/Females	9 M/6 F	7 M	15 M	5 M/1 F	3 M/2 F
Genetics	NA	7/7 X-linked	15/15 X-linked	6/6 AR	3/5 AR
					2/5 X-linked

Abbreviations: BM, bone marrow; MSC, mesenchymal stromal cell; HD, healthy donor; CGD, chronic granulomatous disease; WAS, Wiskott-Aldrich syndrome; SCID, severe combined immune deficiency; ADA, adenosine deaminase deficiency; RAG, recombination activating gene; m, months; y, years; pts: patients; NA, not applicable; NS, not significant; M, males; F, females; NA, not available; AR, autosomal recessive.

HD-derived peripheral blood mononuclear cells (PBMCs) were isolated from buffy-coats obtained from the Unit of Immuno-Hematology and Transfusion Medicine, Bambino Gesù Children’s Hospital, Rome, using a Ficoll-Paque density gradient.

### Isolation and expansion of BM-derived PID- and HD-MSCs

BM-mononuclear cells (MNCs) were isolated from BM of both PID patients and HDs by density gradient centrifugation (Ficoll 1.077 g/ml; Lympholyte, Cedarlane Laboratories Ltd., The Netherlands) and plated in non-coated 75–150 cm^2^ tissue culture flasks (BD Falcon, NJ) at a density of 160,000/cm^2^ in complete culture medium: DMEM (Euroclone, Milan, Italy) supplemented with 10% fetal bovine serum (FBS; Gibco, Life Technologies Ltd, Paisley, UK) penicillin (50 U/ml), streptomycin (50 mg/ml) (P/S) and L-glutamine (2 mM) (L-Glu, Euroclone). In some cases, PID-MSCs were also isolated from the CD34- fraction of BM, after CD34+ purification, as recently described by Ingo *et al*.^44^. After 48-hour culture, non-adherent cells were removed and culture medium was replaced twice a week. MSCs were harvested, after reaching ≥80% confluence, using Trypsin (Euroclone), and were propagated at 4,000 cells/cm^2^ until reaching replicative senescence.

### Characterization of BM-derived PID- and HD-MSCs

#### CFU-F ability and proliferative capacity

Fibroblast colony-forming unit (CFU-F) formation was assessed by examining the cultures at day +7; the clonogenic efficiency was calculated as the number of colonies per 10^6^ MNCs initially seeded for HD-, CGD-, WAS- and ADA-MSCs. Population doublings (PDs) were determined at each passage for each MSC sample by using the formula log_10_(N)/log_10_(2) where N means cells harvested/cells seeded; PDs were calculated for HD-, CGD-, WAS-, ADA- and SCID-MSCs; results were expressed as PD from passage (P) 1 to P5.

#### Senescence assay

MSCs were maintained in culture until reaching replicative senescence. The number of passages in culture before observation of senescence was defined as the life span of *in vitro* MSCs. Cells were closely monitored during senescence for up to 6–8 weeks before interrupting the culture. Senescence of MSCs was also assessed by staining with a senescence β-galactosidase (SA-β-gal) Staining Kit (Cell Signaling Technology, Danvers, MA), according to manufacturer’s instructions.

#### Immunephenotype by FACS

MSC were phenotypically characterized by flow-cytometry at P2-P3 to evaluate the presence of the surface markers CD13, CD90 and CD105 and the absence of CD34, CD45 and CD80, using fluorescein isothiocyanate (FITC) or phycoerythrin (PE)-conjugated monoclonal antibodies (all from BD PharMingen, San Diego, CA). To analyze peripheral blood HD-derived monocytes and B lymphocytes, anti-CD1a allophycocyanine (APC), -CD14 FITC FITC and PE, -HLA-DR FITC, CD86 peridin chlorophyll protein-cyanine (PerCp-Cy 5.5), -CD19 APC-H7, -CD27 PE, -CD38 phycoerythrin–cyanine 7 (PE-Cy7), -IgM Alexa Fluor 647 (BD PharMingen, San Diego, CA) -conjugated monoclonal antibodies were utilized. Analysis of cell population was performed by means of direct immunofluorescence with a FACSCanto flow-cytometer (BD PharMingen); calculations were performed with the FACSDiva software (Tree Star, Inc. Ashland, OR).

#### Western-Blot (WB) for Gp91phox and WAS protein (WASp) expression

Cells were lysed in RIPA buffer (Thermo Scientific, Rockford, IL) containing phosphatase (PhosSTOP phosphatase inhibitor cocktail tables, Roche) and protease inhibitors (Protease inhibitor cocktail tables; Roche) or in an *in*-*house* prepared Laemly Buffer (WASp) for 30 minutes on ice. Lysates were collected and stored at −20 °C. Lysates and supernatants were separated on 10% Mini-Protean TGX Gels (BioRad) with TRIS buffer and transferred into Trans-Blot Turbo Transfer membranes (BioRad). Membranes were blocked for half an hour with Blocking Buffer (TBS containing 0.05% Tween 20 and 5% low fat milk). Membranes were then incubated with primary antibody diluted in blocking buffer for 1 hour at room temperature or overnight at 4 °C. The following antibodies were used: mouse anti-human NOX2 (1:500; Sanquin Blood Supply Foundation), mouse anti-tubulin (1:3000, Sigma), rabbit anti-Human WASP (1:500; Clone H250, Santa Cruz) and mouse anti-Human GAPDH (1:500; MAB374, Millipore). After 3 washing steps, membranes were incubated with horseradish peroxidase-linked secondary antibody diluted in blocking buffer for 1 hour at room temperature (goat anti-mouse or anti-rabbit immunoglobulins/HRP, 1:3000, Dako Denmark A/S). After 3 further washing steps, proteins of interest were detected using Peroxidase substrates (GE Healthcare Life Sciences).

#### ADA enzymatic activity

ADA enzymatic activity was analyzed as reported by Carlucci *et al*.^45^ in ADA-MSCs (3 patients) and HD-MSCs (3 HDs).

### *In vitro* T-cell proliferation assay with phytohemagglutinin

PBMCs were purified by conventional Ficoll separation from heparinized samples obtained from HDs. Cells were processed and used immediately after collection. Before MSC:PBMC co-culture, MSCs were stimulated with IFNγ (10 ng/ml; R&D Systems, Minneapolis, MN) and TNFα (15 ng/ml; R&D Systems) for 18 hours; thereafter, cells were washed twice with PBS and medium was replaced. PBMC proliferation either in the presence or in the absence of MSCs was evaluated after stimulation with phytohemagglutinin (PHA-P 4 μg/ml; Sigma-Aldrich, St Louis, MO) in flat-bottom 96-well tissue culture plates (BD Falcon) containing RPMI 1640 medium (Gibco, Life Technologies Ltd, Carlsbad, CA) supplemented with 10% FBS. Briefly, after MSC seeding and pre-stimulation with pro-inflammatory cytokines, 1 × 10^5^ PBMCs per well were added at final MSC:PBMC ratios 1:2 and 1:10, with PHA. After 3 days, co-cultures were pulsed with ^3^H-thymidine (1 μCi/well, specific activity 6.7 Ci/mmole, Perkin Elmer, Waltham, MA) and cells were harvested 18 hours later. ^3^H-thymidine incorporation was measured with a Microbeta Trilux 1450 instrument (Perkin Elmer). Results were expressed as mean percentage of PBMC proliferation (from 9 different HDs); we referred to PBMC proliferation alone (in the absence of MSCs) as 100% and this percentage was used to normalize PBMC proliferation in the presence of MSCs. All experiments were performed in triplicate in an allogeneic setting (*i*.*e*. PID-MSCs/HD-PBMCs and HD-MSCs/HD-PBMCs).

### *In vitro* B-cell proliferation assay with CpG

Also in this set of experiments, MSCs were pre-stimulated with pro-inflammatory citokines (IFNγ 10 ng/ml and TNFα 15 ng/ml) for 18 hours in order to maximize their immune-regulatory effect. HD-PBMCs (2 × 10^5^), labeled with 0.1 mg/ml of 5-chloromethylfluorescein diacetate (CellTracker CMFDA; Molecular Probes), were seeded on 96-well plates pre-coated or not with 2 × 10^4^ MSCs/well in RPMI 1640 (Gibco BRL, Life Technologies), 10% FBS, penicillin (50 U/ml), streptomycin (50 mg/ml) and L-glutamine (2 mM) (Euroclone), supplemented or not with CpG-ODN (2.5 mg/ml; Hycult Biotechnology, The Netherlands). Cell proliferation and phenotypic analysis was performed by flow-cytometry using a FACSCanto Flow Cytometer (BD Biosciences) after 7-day co-culture.

### Effect of MSCs on monocyte maturation

Monocytes were purified from HD-PBMCs by magnetic activated cell sorting (MACS) using CD14 microbeads (Miltenyi Biotec GmbH, Bergisch Gladbach, Germany), followed by MACS LS column separation according to the manufacturer’s recommendations. To analyze comparatively the effect of PID- and HD-MSCs on monocytes, 7-day cultures of 2 × 10^5^ freshly isolated monocytes were plated in a transwell insert (pore size 0.4 µM, Corning, Inc., Lowell, MA) on 24-well plates pre-coated with MSCs in ratios MSC:Monocyte 1:10–1:20–1:50. RPMI 1640 (Gibco BRL, Life Technologies), 10% FBS, penicillin (50 U/ml), streptomycin (50 mg/ml) and L-glutamine (2 mM; Euroclone), supplemented with GM-CSF (100 ng/ml) and IL-4 (25 ng/ml; Life Technologies) was used as medium for the co-culture. The medium was refreshed twice (day 3 and day 5), and after 7 days of culture, monocytes were harvested and analyzed by flow-cytometry.

### Measurement of cytokines by ELISA

Concentration of IL-2, IL-6, IL-10, TGFβ and IFNγ in supernatants of MSC/PBMC co-cultures in the presence of PHA was measured by means of commercially available ELISA kits (Nacka strand, Sweden) after 72 hours incubation. Plates were read at 405 nm through an Envision Multilabel Reader (Perkin Elmer).

### Toll-like Receptor (TLR) priming of MSCs

LPS (10 ng/ml, Sigma-Aldrich, St. Louis, MO) and poly (I:C) (1 µg/ml, Sigma-Aldrich) were used as agonists for TLR4 and TLR3, respectively, in order to activate MSCs. Briefly, 1 × 10^5^ MSCs were plated on 6-well plates and grown to 60–70% confluency in complete culture medium prior to the start of the experiments. TLR-agonists were added to fresh medium for 1 hour; thereafter, MSCs were washed twice in complete medium without the TLR-agonists and incubated for 18 hours before collecting their RNA for gene expression assay.

### PID-MSC differentiation capacity into osteoblasts and adipocytes

The osteogenic and adipogenic differentiation capacity of MSCs was assessed at P2–P4, as previously described^29^. To detect osteogenic differentiation, cells were stained for alkaline phosphatase (AP) activity using Fast Blue (Sigma-Aldrich) and for calcium deposition with Alizarin Red (Sigma-Aldrich). Adipogenic differentiation was evaluated through the morphological appearance of fat droplets stained with Oil Red O (Sigma-Aldrich).

### Reverse transcriptase-quantitative polymerase chain reaction (RT-qPCR) for osteogenic/adipogenic, immunomodulatory and disease-associated markers

RNA was extracted with TRIzol (Life Techologies) according to the manufacturer’s instructions. RNA was reverse transcribed into complementary DNA (cDNA) by Super Script II Reverse Transcriptase (Life Techologies), according to the manufacturer’s instructions. qPCR assays were performed with TaqMan gene expression assays (Applied Biosystems, Life Technologies, Carlsbad, CA), using the following TaqMan probes: peroxisome proliferator-activated receptor-γ (PPARγ, Hs00234592_m1, as adipogenic marker), bone morphogenetic protein 2 (BMP2, Hs00154192_m1, as *early* bone differentiation marker), Runt-related transcription factor 2 (RUNX2, Hs01047973_m1, as *early* bone differentiation marker), Alkaline phosphatase liver/bone/kidney (ALPL, Hs01029144_m1, as *late* bone differentiation marker), secreted phosphoprotein 1 (SPP1, Hs00959010_m1, as *late* bone differentiation marker), Toll-like receptor 3 (TLR3, Hs01551078_m1), Toll-like receptor 4 (TLR4, Hs00152939_m1), indoleamine 2,3-dioxygenase 1 (IDO, Hs00984148_m1), hepatocyte growth factor (HGF, Hs00300159_m1), Regulated upon Activation, Normal T-cell Expressed and Secreted (RANTES, Hs00982282_m1), C-X-C motif chemokine ligand 9 (CXCL9, Hs00171065_m1), C-X-C motif chemokine ligand 10 (CXCL10, Hs00171042_m1), cytochrome b-245 beta chain (CYBB, Hs00166163_m1), Wiskott-Aldrich syndrome (WAS, Hs00166001_m1), adenosine deaminase (ADA, Hs01110945_m1) and glyceraldehyde-3-phosphate dehydrogenase (GAPDH, Hs99999905_m1), used as housekeeping gene (Applied Biosystems, Life Technologies), with an Applied Biosystems 7900 system. qPCR was carried out in duplicate for each data point. The relative quantification of the gene expression was determined normalizing the data of the gene to GAPDH housekeeping gene and using the 2^−ΔΔCT^ method.

### Statistical analysis

Data were reported as mean ± standard error (SEM) of the mean. All experiments were performed in triplicate. Statistical significance was determined using the Student’s *t*-test. P values lower than 0.05 were considered to be statistically significant (*p < 0.05; **p < 0.01; ***p < 0.001).

## Electronic supplementary material

Supplementary Figure 1 and 2
