# Supplementary material for: Biological and functional characterization of bone marrow-derived mesenchymal stromal cells from patients affected by primary immunodeficiency
Source: Sci Rep. 2017 Aug 15;7:8153. doi: 10.1038/s41598-017-08550-5 (PMC5557950; doi:10.1038/s41598-017-08550-5)
Supplement: Supplementary file 1 — Supplementary Figure 1 and 2 [file 41598_2017_8550_MOESM1_ESM.doc]

**Biological and functional characterization of bone marrow-derived mesenchymal stromal cells from patients affected by primary immunodeficiency**

***Nadia Starc,^1,2^ Daniela Ingo,^3^ Antonella Conforti,^1^ Valeria Rossella,^3^ Luigi Tomao,^1^ Angela Pitisci,^1^ Fabiola De Mattia,^3^ Immacolata Brigida,^3^ Mattia Algeri,^1^ Mauro Montanari,^1^ Giuseppe Palumbo,^1,2,4^ Pietro Merli,^1^ Paolo Rossi,^2,4^ Alessandro Aiuti,^3,5^ Franco Locatelli^1,6*^ and Maria Ester Bernardo^1,3*^***

^1^Department of Pediatric Hematology/Oncology, IRCCS Bambino Gesù Children’s Hospital, Rome

^2^Department of System Medicine, University of Rome "Tor Vergata", Rome

^3^San Raffaele Telethon Institute for Gene Therapy, SR-TIGET; Pediatric Immunohematology, San Raffaele Scientific Institute, Milan

^4^University Department of Pediatrics, Unit of Immune and Infectious Diseases, IRCCS Bambino Gesù Children’s Hospital, Rome

^5^Vita-Salute San Raffaele University, Milan

^6^Department of Pediatrics, University of Pavia, Pavia, Italy.

* Co-corresponding and co-last authors

**Supplementary Figure 1. Immuno-phenotype of culture-expanded CGD-, ADA- and SCID-MSCs. Histograms are similar in terms of forward scatter (FSC) and side scatter (SSC), as well as of surface marker expression (positive for CD13, CD90, CD105 surface antigens and negative for CD34, CD45 and CD80 molecules).**

**
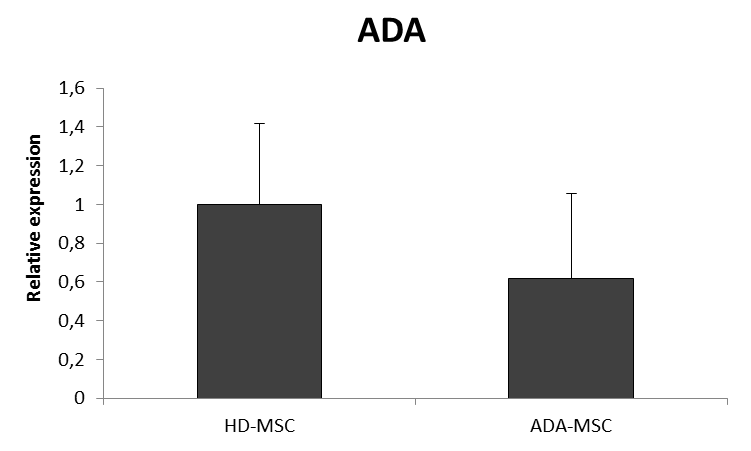
**

**Supplementary Figure 2.** Gene expression of ADA on HD- and **ADA-MSCs**. mRNA levels were quantified by the 2^-ΔΔCt^ method after normalization with respect to GAPDH. Results are expressed as fold-change relative to HD-MSCs. Each bar represents the mean ± SEM of multiple experiments (each point were performed in duplicate and repeated independently at least 3 times).

| **SENESCENCE** | **MEAN**  **PASSAGE** | **RANGE**  **PASSAGE** | **COMPARISON BETWEEN**  **PID-MSC AND HD-MSC** |
| --- | --- | --- | --- |
| HD-MSC | 11 | 9 - 13 | NA |
| CGD-MSC | 9 | 8 - 10 | P= NS |
| WAS-MSC | 12 | 10 - 14 | P= NS |
| ADA-MSC | 11 | 10 - 12 | P= NS |
| SCID-MSC | 10 | 9 - 11 | P= NS |

**Supplementary Table 1. Study of the senescence phase of PID-MSCs as compared with HD-MSCs. Abbreviations: MSC, mesenchymal stromal cell; HD, healthy donor; CGD, chronic granulomatous disease; WAS, Wiskott-Aldrich syndrome; ADA, adenosine deaminase deficiency; SCID, severe combined immune deficiency; NA, not applicable; NS, not significant.**
